# Temporal context and conditional associative learning

**DOI:** 10.1186/1471-2202-11-45

**Published:** 2010-03-30

**Authors:** Oussama H Hamid, Andreas Wendemuth, Jochen Braun

**Affiliations:** 1 Department of Cognitive Biology, Institute of Biology, Otto-von-Guericke University, Leipziger Str. 44, 39120 Magdeburg, Germany; 2 Department of Cognitive Systems, Institute of Electronics, Signal Processing, and Communications, Otto-von-Guericke University, Universitätsplatz 2, 39106 Magdeburg, Germany

## Abstract

**Background:**

We investigated how temporal context affects the learning of arbitrary visuo-motor associations. Human observers viewed highly distinguishable, fractal objects and learned to choose for each object the one motor response (of four) that was rewarded. Some objects were consistently preceded by specific other objects, while other objects lacked this task-irrelevant but predictive context.

**Results:**

The results of five experiments showed that predictive context consistently and significantly accelerated associative learning. A simple model of reinforcement learning, in which three successive objects informed response selection, reproduced our behavioral results.

**Conclusions:**

Our results imply that not just the representation of a current event, but also the representations of past events, are reinforced during conditional associative learning. In addition, these findings are broadly consistent with the prediction of attractor network models of associative learning and their prophecy of a persistent representation of past objects.

## Background

Conditional associative tasks probe the ability of primates to learn arbitrary sensorimotor mappings [1,2]. Typically, the experimental design takes a set of visual stimuli from the same category and maps them randomly onto a set of motor responses. Subjects learn by trial and error which response produces the reward in the case of each stimulus (e.g., if stimulus A, then response X secures the reward). As all stimuli are potentially associated with reward, the subject cannot simply learn stimulus-reward associations. Instead, subjects must link each stimulus to the specific response that ensures the reward in each case. This requires not only stimulus recognition and response selection, but also keeping track of (at least some of) the stimulus-response pairings already tried and the outcomes obtained. Depending on the size of the stimulus set, this may generate a considerable memory load.

Studies with behaving primates reveal an extensive network of brain areas underlying conditional associative tasks [3,4]. The associative link between visual object recognition, subserved by inferior temporal cortex [5-8], and response selection, mediated by prefrontal and premotor cortex [9-11] does not, however, appear to involve a direct interaction of these brain areas [12]. Instead, conditional associative learning seems to rely on indirect pathways through the striatum [13-16] and the medial temporal lobe [17-20].

With more extensive stimulus sets, conditional associative tasks are suitable also for human observers. Functional imaging studies confirm that such tasks involve a similar network of prefrontal, parietal, and striatal areas in the human brain as in the brain of non-human primates [21-24].

Attractor network models of associative learning [25,26] predict that memories should be shaped by the order in which different events are rehearsed. The reason is that the neural representation of an event class - its attractor state - should linger even after a triggering event has passed. Due to this reverberatory 'delay activity', events that occur consistently in a particular temporal order should eventually become subsumed under the same event class in associative memory. Importantly, it is the consistent temporal order, not mere temporal proximity, that should lead to these expanded memory representations.

Behavioral results from human observers are consistent with the idea that temporal order shapes associative learning [27,28]. For example, observers suffer in their ability to distinguish two face images after viewing image sequences in which the face identity changes as the head rotates [29]. Apparently, the correlated appearance over time leads observers to classify the two faces as the same person. Similarly, human observers come to classify two distinct objects as "similar" when they have repeatedly viewed a series of intermediate objects [30]. Importantly, the distinct objects become associated only if the intermediate objects were presented in a systematic order, starting with the most similar and ending with the most dissimilar to the initial object. Once again, it appears as if perceiving objects in a consistent temporal order would merge their representations in associative memory. More generally, temporal order effects are well documented for serial reaction time tasks [31-33] and serial visual search tasks [34,35] with human observers, as well as for serial button press tasks with non-human primates [36,37].

More direct evidence for an effect of temporal order on associative memory comes from electrophysiological recordings in behaving non-human primates. When monkeys are trained to perform a delayed match-to-sample tasks, neurons in the inferior temporal cortex exhibit stimulus-selective activity during the delay period [38], as a consequence of having formed a long-term associative memory of a stimulus [39,40]. When different sample stimuli are presented in a consistent order over successive trials, some neurons develop a task-irrelevant selectivity for successive sample pairs [40]. In monkeys trained to associate different objects that are presented successively (paired-associate tasks), delay activity for the first object and neuronal selectivity for the pairs become evident concurrently and in the same neurons [41,42] (see also [43]). These observations directly link consistent temporal order, the presence of 'delay activity', and the merging of associative memory representations.

Here, we introduce a novel approach to studying the effect of temporal order on associative learning with human observers. Our approach is patterned on established paradigms of conditional associative learning and, unlike the previous studies mentioned above, does not involve sequences of self-similar images (e.g., incrementally rotated [29] or morphed faces [30]). This choice was motivated by several considerations. Firstly, we wanted to stay as close as possible to the behavioral situation of the non-human primate studies in which temporal order effects were first described [39,40]. Secondly, we wanted more freedom to manipulate temporal order than was possible with self-similar images. Thirdly, we wanted to conceal the presence of temporal order from observers, in order to minimize complications arising from cognitive strategies that often beset human studies.

**Figure 1:**
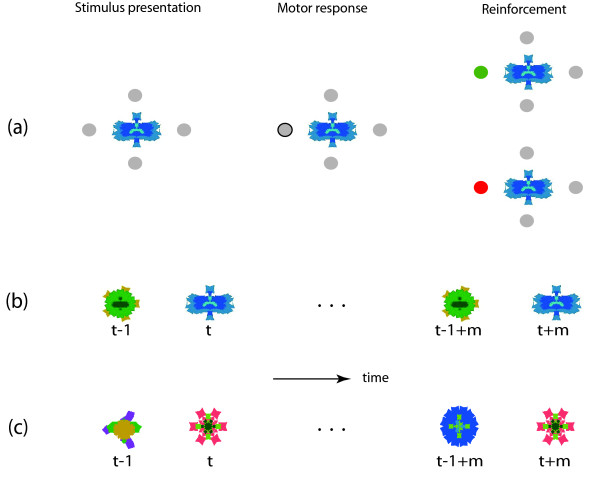
**Experimental design (schematic)**. Each trial comprises three phases: stimulus presentation, motor response, and reinforcement. Firstly, a fractal object appears (center), surrounded by four response options (grey discs). Secondly, the observer reacts by pressing the key that corresponds to one response option (outlined disk). Thirdly, a color change of the chosen option provides reinforcement (green if correct, red if incorrect). (**b**) Object sequence *with *temporal context. Target objects recur every 2 to 48 trials. Thus, successive trials always present different objects. A consistent temporal context is created by the fact that each target object (*e.g.*, trials *t *and *t *+ *m*) is preceded consistently by a specific (other) object (trials *t - *1 and *t *+ *m - *1). (**c**) Object sequence *without *temporal context. Each time an object appears (trials *t *and *t *+ *m*), it is preceded by a different object (trials *t - *1 and *t *+ *m - *1).

Specifically, our observers viewed highly distinguishable, fractal objects and learned to select one of four possible motor responses for each object. Some objects were consistently preceded by specific other objects, while other objects lacked such a predictive temporal context (Figure 1). Our aim was to keep observers engaged in the immediate task (learning visuo-motor associations) and to discourage as far as possible any performance strategies relying on temporal context. For this reason, we intermixed (in most experiments) visual objects with and without temporal context and ensured that knowledge of temporal context was not necessary for accurate performance. Our results show that observers expended comparable attention and/or memory resources on objects with and without temporal context, confirming that observers applied comparable learning strategies in both cases.

To better interpret our behavioral results, we devised a model of reinforcement learning [44] for our behavioral paradigm. In this model, response choice is based on multiple action values, some attaching to the object of the current trial and others attaching to objects of preceding trials. As a consequence, our model exhibits a similar dependence on temporal context as do human observers.

In summary, we have studied the effect of temporal context on conditional associative learning. Our behavioral situation is based on non-human primate paradigms but conceals the presence or absence of temporal context from human observers. We believe that this is a promising approach to testing the predictions of attractor theory of associative learning with human observers.

## Results

### Behavioral results

To ascertain whether temporal context influences the process of associative learning (or not), we conducted five behavioral experiments. In all experiments, observers learned to recognize and to classify fractal objects [39]. The objects were initially unfamiliar but highly distinguishable. For each object, observers were asked to learn the 'correct' motor response (one of four) associated with this object. After the observer's choice, the response was identified as 'correct' or 'incorrect'. Most objects recurred multiple times during the session ('recurring objects'), providing ample opportunity for learning by trial and error. Some experiments also used 'one-time objects', which appeared only once.

A trial consisted of the presentation of one object, the observer's response to that object, and reinforcement (Figure 1a). Trial sequences differed in length (56 to 336 trials) and in the number of recurring objects (8 to 16 objects), resulting in learning situations of greatly varying difficulty. Each trial sequence used new and unfamiliar objects, forcing observers to relearn the objects each time.

**Figure 2:**
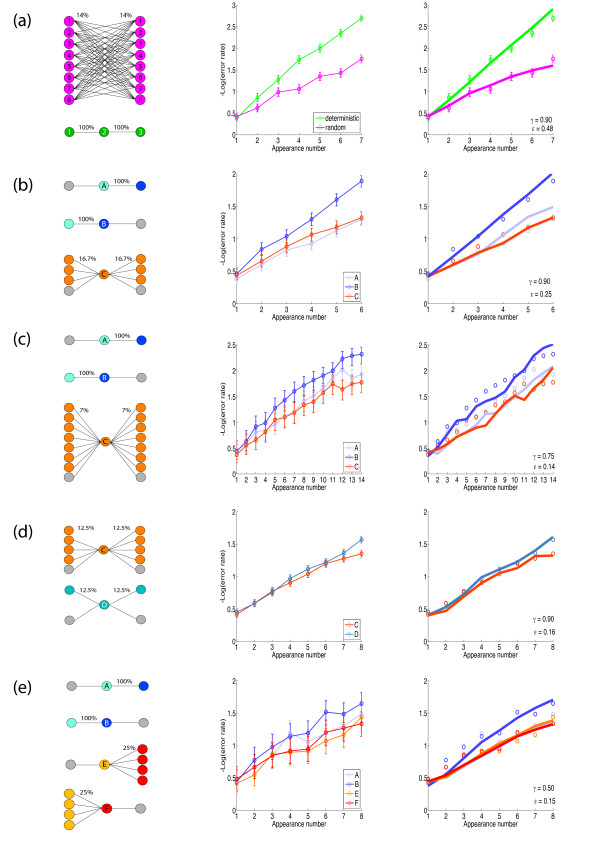
**Behavioral and modeling results**. For each of five experiments, temporal context, behavioral performance, and predicted performance are shown (left, middle, and right columns, respectively). Trial sequences were composed of 'recurring objects' (types A-F) distinguished by their temporal context. Error bars refer to the 95% confidence intervals (α = 0.05) for binomially distributed data. In **(b)-(e)**, recurring objects were intermixed with 'one-time objects'. Type A objects were preceded by a one-time object and followed by one particular other recurring object (probability 100%). Type B objects were preceded by one particular other recurring object (probability 100%) and followed by a one-time object. Type C objects were preceded (followed) by one-time objects (probability 50%) and by each of several other recurring objects (cumulative probability 50%). Type D objects were preceded (followed) by one-time objects (probability 50%) and by one particular other recurring object (probability 50%). Type E objects were preceded by a one-time object and followed by each of four other recurring objects (probability 25%). Type F objects were preceded by each of four other recurring objects (probability 25%) and followed a one-time object. The relative informativeness of the temporal contexts is given in Table 1. **(a) **Eight objects appeared seven times each, in either deterministic or random sequences. In deterministic sequences, each object was preceded (followed) seven times (100% probability) by one particular of the other seven objects. In random sequences, each object was preceded (followed) once (14% probability) by each of the seven other objects. **(b) **Eight recurring objects (2 type A, 2 type B, and 4 type C) appeared six times each, intermixed with one-time objects. **(c) **Sixteen recurring objects (4 type A, 4 type B, and 8 type C) appeared 14 times each. **(d) **Ten recurring objects (5 type C and 5 type D) appeared eight times each. **(e) **Sixteen recurring objects (4 each of types A, B, E, and F) appeared 8 times each.

Pilot experiments established that human observers consistently approach ceiling performance (*P *= 100% correct) if the trial sequence is sufficiently long. A convenient performance measure is therefore the negative logarithm of the distance to ceiling performance (*- *log_2 _[1 *- P *]). In terms of this measure, performance improves almost linearly with every object appearance (Figure 2).

**Table 1:** Informativeness of temporal context.

	Object type					
	
Experiment	A	B	C	D	E	F
1		100%	2.0%			
2	0%	100%	2.8%			
3	0%	100%	0.5%			
4			1.5%	20.3%		
5	0%	100%			0%	0%

Mutual information between predecessor object and correct response of current object, as a percentage of 2 *bit*, the mutual information between object and correct response (equations 10 and 11 in Methods section).

The 'correct' response of each trial was determined completely by the object of that trial, which thus provided 2 bits of information. However, the object of the preceding trial was sometimes informative as well. This 'temporal context' information was redundant and, except in experiment 1, observers appeared unaware of its availability. When asked about their behavioral strategy, observers indicated consistently that they had concentrated their efforts on the current object.

The informativeness of the object in the previous trial (about the correct response in the current trial) was quantified as percentage of informativeness of the current object (see section entitled "Mutual information" in Methods). Thus, the informativeness of this temporal context ranged from 0% to 100% (Figure 1bc). Table 1 summarizes the informativeness of the various temporal contexts employed in experiments 1 to 5. The level of significance adopted for all the statistical comparisons reported here was set at *p *< 0.05.

#### Experiment 1

Eight fractal objects appeared seven times each, in either a deterministic or a random sequence (Figure 2a). Both types of sequence were 56 trials long. In deterministic sequences, each object was preceded (followed) seven times (100% probability) by one particular of the other seven objects. In random sequences, each object was preceded (followed) once (14% probability) by each of the seven other objects. Accordingly, the temporal context of deterministic and variable sequences was, respectively, 100% and 2% as informative about the correct response as the current object itself (see Table 1 and "Mutual information" in Methods). Observers quickly understood the existence and nature of the two types of sequences (even though the instructions had been silent on this point). Accordingly, it seemed likely that observers applied a different learning strategy in each case. The average results for 10 observers are presented in (Figure 2a). Post hoc *t*-tests revealed that learning was significantly faster in deterministic than in variable sequences (*t*(239) = 2.3, *p *< 0.03), exhibiting initial learning rates of 0.13 and 0.04 *bit *per appearance, respectively (average across subjects). While this difference may have been due to the disparate temporal contexts, it could also have reflected differential allocation of attentional and/or memory resources on the part of the observers.

#### Experiment 2

To ascertain whether learning rate depends on the temporal context of individual objects, we created sequences that intermixed 'recurrent objects' with different temporal contexts as well as 'one-time objects'. In this situation, observers are less likely to allocate differential attentional and/or memory resources to different object types.

Eight recurring objects appeared six times each, intermixed with 24 one-time objects, in sequences of 72 trials (Figure 2b). Each of two type A recurring objects was preceded by a one-time object and followed consistently (100% probability) by one particular other recurring object (type B). Each of two type B recurring objects was consistently (100% probability) preceded by one particular other recurring object (type A) and followed by a one-time object. Each of four type C recurring objects was preceded (followed) once (16.7% probability) by each of the three other recurring objects (type C) and three times (50% probability) by a one-time object.

The temporal context of type A, B, or C objects was, respectively, 0%, 100%, and 2.8% as informative as the object itself (Table 1). The average results for 8 observers are presented in (Figure 2b). Beginning with the second appearance, learning was significantly faster for objects with more informative (type B) than with less informative (type C, type A) temporal contexts (type B vs. type A: *t*(227) = 3.1, *p *< 0.01; type B vs. type C: *t*(227) = 2.9, *p *< 0.01). The initial average rates of learning were 0.12, 0.05, and 0.03 *bit *per appearance for type B, C, and A objects, respectively.

#### Experiment 3

The previous experiment demonstrated that learning rate depended on the temporal context of each object in a sequence. To ascertain whether this effect would persist with a higher memory load, we conducted a similar experiment with 16 (rather than 8) recurrent objects. To increase the sensitivity of the measurements, each recurrent object appeared 14 (rather than 6) times.

Sixteen recurring objects appeared 14 times each, intermixed with 112 one-time objects, in sequences of 336 trials (Figure 2c). Each of four type A recurring objects was preceded by a one-time object and followed consistently (100% probability) by one particular other recurring object (type B). Each of four type B recurring objects was consistently (100% probability) preceded by one particular other recurring object (type A) and followed by a one-time object. Each of eight type C recurring objects was preceded (followed) once (7% probability) by each of the seven other recurring objects (type C) and seven times (50% probability) by a one-time object.

The temporal context of type A, B, or C objects was, respectively, 0%, 100%, and 0.5% as informative as the current object (Table 1). The results of 5 observers are summarized in (Figure 2c). Beginning with the fifth appearance, learning was significantly faster for objects with more informative (type B) than with less informative (type C, type A) temporal contexts (type B vs. type A: *t*(59) = 2.2, *p *< 0.04; type B vs. type C: *t*(59) = 2.7, *p *< 0.01). The initial average rates of learning were 0.10, 0.06, and 0.05 *bit *per appearance for type B, C, and A objects, respectively.

#### Experiment 4

Previous experiments compared temporal contexts that were either maximally or minimally informative. In a further experiment, we compared temporal contexts with an intermediate degree of informativeness. To this end, we presented each object in several contexts, only some of which were informative.

Ten recurring objects appeared 8 times each, intermixed with 40 one-time objects, in sequences of 120 trials (Figure 2d). Each of five type C recurring objects was preceded (followed) once (12.5% probability) by each of the four other recurring objects (type C) and four times (50% probability) by a one-time object. Each of five type D recurring objects was preceded (followed) four times (50% probability) by one particular other recurring object (type D) and four times by a one-time object.

The temporal context of a type C or D object was, respectively, 1.5% and 20.3% as informative as the object itself (Table 1). Figure 2d summarizes the results of 10 observers. Initial learning rates were comparable for type C and D objects (0.06 and 0.07 *bit*, respectively), although type D objects gained a modest advantage after further appearances. Only at the eighth (last) appearance was there a significant difference in learning between type D and type C objects (*t*(689) = 2.2, *p *< 0.03). The fact that observers failed to learn type D objects more rapidly than type C objects suggests that partially informative temporal contexts do not accelerate learning. Of course, it remains possible that learning would be accelerated by temporal contexts that are, say, 75% informative (*i.e*., more than 20%, yet less than 100% informative).

#### Experiment 5

To allay any concern that observers might have allocated differential attention/memory resources to different object types, we conducted one further experiment on this point. Specifically, we presented recurrent objects in ordered pairs, some objects serving consistently as first members and others consistently as second members of these pairs. In some pairs (type A and type B objects), the first members were informative about the second members whereas, in other pairs (type E and type F objects), the first members were uninformative about the second members. If consistent object pairings had attracted additional attention/memory resources to the second member of each pairing, then this should have been true for both types of pairs, resulting in faster learning of both type B and type F objects. Sixteen recurring objects appeared 8 times each, intermixed with 64 one-time objects, in sequences of 192 trials (Figure 2e). Each of four type A objects was preceded by a one-time object and followed consistently (100% probability) by one particular other recurring object (type B). Each of four type B objects was preceded consistently (100% probability) by one particular other recurring object (type A) and followed by a one-time object. Each of four type E objects was preceded by a one-time object and followed twice (25% probability) by each of four other recurring objects (type F). Each of four type F objects was preceded twice (25% probability) by each of four other recurring objects (type E) and followed by a one-time object. The temporal context of type A, B, E, or F objects was, respectively, 0%, 100%, 0%, and 0% as informative as the object itself. Figure 2e summarizes the results of 5 observers. Beginning with the seventh appearance, learning was significantly faster for objects with more informative (type B) than less informative (type A, type E, and type F) temporal contexts (type B vs. type A: *t*(29) = 2.24, *p *< 0.04; type B vs. type E: *t*(29) = 4.5, *p *< 0.001; type B vs. type F: *t*(29) = 2.8, *p *< 0.01). The initial average rates of learning were 0.15, 0.09, 0.06, and 0.09 *bit *per appearance for type B, type A, type E, and type F objects, respectively. In short, only informative temporal context led to faster learning. Merely presenting objects as consistent pairs (without the first object being informative about the second) did not accelerate learning. This failure shows conclusively that accelerated learning is due to informative temporal context, not to additional attention/memory resources.

#### One-time objects

As learning progresses, observers tend to react faster to recurring objects (whether with or without temporal context). However, reaction times to one-time objects remained consistently slow throughout the trial sequence, suggesting that observers do try to learn (i.e., expend attentional and memory resources) even on one-time objects.

To assess the predictive value, if any, of one-time objects, we compared performance and reaction time for type C objects that followed a one-time-object and for (the identical) type C objects that followed other type C objects (experiments 2, 3, and 4). We found no significant difference in either performance or reaction time between type C objects in these different contexts.

It remains possible that the (comparatively poor) performance on type A objects may have benefitted from their consistent temporal association with one-time objects. However, our sequences lacked a suitable control object so that we could not test this possibility.

#### Summary

An 'ideal learner' accumulates information about the correct response to a particular object at an initial average rate of 0.5 *bit *per appearance (see below). Human observers performed substantially less well, accumulating on average 0.09 and 0.07 *bit *during the initial appearance of a recurrent object in experiments 1 and 2 (memory load 8 objects), 0.07 *bit *in experiment 4 (10 objects), and 0.07 and 0.1 *bit *in experiments 3 and 5 (16 objects). These values represent learning in the absence of any temporal context provided by previous objects.

**Figure 3:**
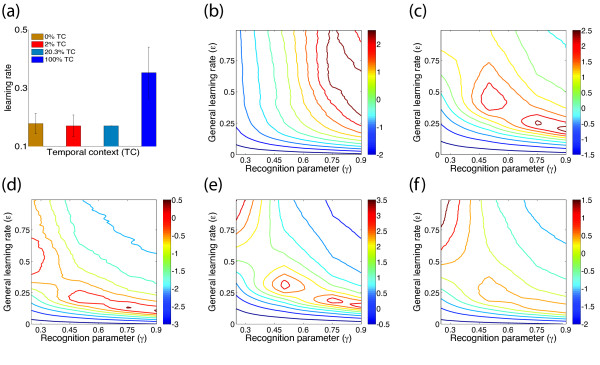
**Actual learning rates and estimated parameters**. **(a) **Acceleration of learning during the initial appearance of objects due to different degrees of temporal context. In the presence of a fully predictive temporal context, the accumulation of information was accelerated by 0.13 *bit*. Error bars in Figure 3a show the standard deviation across experiments for each object type. Plots **(b)-(f) **show regions of optimal values in the parameter space (ϵ, *γ*), corresponding to the general learning rate and the recognition parameter, respectively. The color scales to the right of each plot refer to the fit quality *f*_*Q *_for each parameter pair (ϵ, *γ*), which was computed as , where  and  are the mean values of performance correct in the *i*-th appearance for human observers and for the model simulations, respectively, and  and  are the corresponding standard deviations. The higher the *f*_*Q *_values, the better the fit between measured and predicted data.

In the presence of temporal context, the accumulation of information was accelerated by 0.13 *bit *during the initial appearance of objects embedded in a fully predictive temporal context (Figure 3a).

### Computational results

#### Basic model, insensitive to context

A simple model for our situation is that response probabilities are modified directly such as to maximize expected reward. For each object *n*, four response probabilities , where *j *∈ {1, ⋯, 4} and  must be learned. When object *n *is observed, action *k *is selected, and reward *r*_*k *_∈ {0, 1} is received, a suitable rule for updating response probabilities is(1)

where *λ *and *μ *are learning rates in the range of [0, 1] and *δ*_*jk *_is the Kronecker delta (which equals 1 if *j *= *k *and 0 if *j *≠ *k*). This rule ensures  and . Choosing *λ *> *μ *makes learning faster in rewarded than in unrewarded trials. Choosing the maximal rates *λ *= *μ *= 1 implements an 'ideal learner'. Note that this simple model ignores temporal context and focuses on the explicit task (associating the current object with the rewarded choice). As a result, this model does not predict any dependence of learning rate on temporal context and therefore does not account for our behavioral results.

#### Extended model, sensitive to context

We now introduce a more elaborate model that is sensitive to temporal context. We choose an indirect actor model that responds probabilistically on the basis of reward expectations.

##### Probabilistic response

The probability of choosing response *k *in trial *t *is(2)

where  is the reward expected from response *k *in trial *t*. The parameter *β *determines whether the model behaves in a more exploratory or a more exploitative manner. We use *β *= 20.

##### Reward expectation

Reward expectations are based on 'action values' that have accumulated for the objects of the current trial, *t*, and the two previous trials, *t - *1 and *t - *2. Each object *x *is associated with 12 action values , where *i *indexes current, next, and after-next trials (*i *∈ {0, 1, 2}) and *j *indexes the response possibilities (*j *∈ {1, ⋯, 4}). In the case of a familiar object, action values reflect past experience as to which responses were rewarded and which unrewarded after the object in question had been observed. In the case of unfamiliar objects, all action values are initialized to 0.

**Figure 4:**
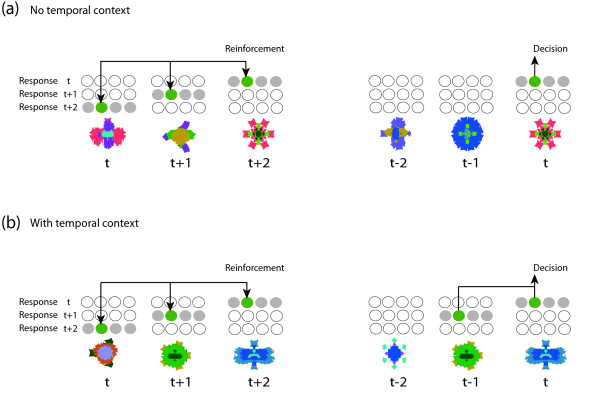
**Reinforcement of action values (schematic)**. Each object is associated with 12 action values. For the object in trial *t*, 4 action values inform the response of the current trial *t*, 4 values concern the response of the next trial *t *+ 1, and the remaining 4 values contribute to the response of the second next trial *t *+ 2. Correspondingly, the response of trial *t *is based on 12 actions values: 4 values of the current object *t*, 4 values of the previous object *t - *1, and 4 values of the pre-previous object *t - *2. Temporal context determines which action values are reinforced consistently. (**a**) In the absence of temporal context, only the current object's action values are reinforced consistently and come to reflect the correct choice. In this case, the decision in trial *t *is based on 4 action values of object *t*. (**b**) In the presence of temporal context, both the current and the previous object's action values are reinforced consistently. Thus, the decision in trial *t *is based on 4 action values of object *t *and 4 action values of object *t - *1.

Specifically, if objects *n"*, *n'*, and *n *appeared in trials *t - *2, *t - *1, and *t*, respectively, and if each object is recognized unambiguously, the reward expectation for response *j *in trial *t *is(3)

combining action values of the current, the previous, and the before-previous objects. Temporal context determines which action values are reinforced consistently and, thus, which values come to indicate the correct response. In the absence of temporal context, only the current object's action values are reinforced consistently and thus become indicative of the correct response (Figure 4). Note that the model does not assume any attenuation of past objects: current, previous, and before previous objects all contribute equally to reward expectation.

##### Action values

Action values are reinforced by a modified Rescorla-Wagner rule [45]. If a response *k *receives a reward  in trial *t*, the prediction error is(4)

and the three action values , , and  associated with action *k *are modified as follows:(5)

where *x *= *n*, *n'*, and *n" *when *i *= 0, 1, and 2 respectively, ϵ is the general learning rate, and  is the specific learning rate of object *x *∈ {*n*, *n'*, *n"*} in trial *t *(see below). Action values associated with other actions *j *≠ *k *remain unchanged.

##### Recognition parameter

Human observers sometimes fail to recognize an object they have seen before. To model this confusion about object identity, we introduce a *recognition parameter γ*, 0 ≤*γ *≤ 1, which parametrizes the extent to which an actual object is recognized as being present. The value of *γ *affects learning in two ways. Firstly, it influences the reward expectation by taking into account not only the objects actually present but also all other objects. As a result, (equation 3) becomes(6)

where  for *i *∈ {0, 1, 2} and *x *∈ {*n*, *n'*, *n"*}. *N *is the total number of objects. Secondly, *γ *< 1 removes some reinforcement from action values of objects actually present and distributes the reinforcement over the action values of all other objects. Accordingly, (equation 5) modifies to(7)

where *i *∈ {0, 1, 2} and *x *∈ {*n*, *n'*, *n"*}.

The recognition parameter *γ *is an admittedly crude way of modeling confusion about object identity. In human observers, one might expect that recognition rates increase with every appearance of a particular object. In our model, the value of *γ *does not reflect this (hypothetical) improvement and remains constant throughout the sequence.

##### Specific learning rates

Specific learning rates reflect how reliably a particular object is associated with the reward and are computed by a Kalman-filter algorithm [46]. Let **x**^(*t*) ^be the augmented stimulus vector of trial *t *which comprises three components for each object *n*_*i *_∈ {*n*_1_, ..., *n_N_*} (one component for each the current, the previous, and the before-previous trial). The values of **x**^(*t*) ^reflect the recognition parameter and differ for present and absent objects in the following manner:(8)

Here, *j *∈ {1, ..., 3N} and *i *= *j *mod *N*.

The specific learning rate of object *x*_*i *_is computed from(9)

where  is a drift covariance matrix that is accumulated iteratively. The iteration algorithm is given in the appendix.

#### Model fitting

In both basic and extended models, response choices depend on 'action values' that are learned by reinforcement. The basic model, in which action values are associated exclusively with the current object, ignores temporal context in choosing the current response. As a result, the basic model does not account for the sensitivity to temporal context exhibited by human observers. Nevertheless, the basic model provides a useful benchmark to which human performance can be compared.

With learning rates set to their maximal values of *λ *= *μ *= 1, the basic model implements an 'ideal learner'. Its average performance increases from 25% correct on the first appearance of an object, to 50%, 75%, and 100% correct on the second, third, and fourth appearance of the object. The combined entropy of response and reward falls from 2.81 *bit *on the first appearance, to 2.16 *bit*, 1.41 *bit*, and 0 *bit *on the second, third, and fourth appearances, respectively.

In the extended model, action choices are influenced equally by three objects: the current, the previous, and the one preceding the previous object. In addition to this sensitivity to temporal context, the extended model also allows for probabilistic object recognition and employs differental learning rates that depend on the reliability of a reward-association [46].

The extended model has two free parameters, namely, the general learning rate ϵ and the recognition parameter *γ *(equation 7). The parameter *β *did not materially affect the results and its value was kept equal to *β *= 20 throughout (equation 2).

The extended model was fit to the behavioral results in the ranges of 0 ≤ ϵ ≤ 1 and 0.25 ≤ *γ *≤ 0.9 (Figure 3). The results of experiment 1 are consistent with a comparatively rapid learning rate of ϵ ≈ 0.48 and a near-perfect recognition probability of *γ *≈ 0.9 (Figure 3b). Apparently, the simple sequence structure facilitated object recognition.

The results of experiments 2, 4, and 5 are consistent with somewhat lower learning rates and reduced recognition probabilities in the range of *γ *= 0.5 to 0.9 (Figures 3cef). The learning rates appear to decrease with increasing object number, with ϵ≈ 0.25 in experiment 2 (8 recurring objects and 24 one-time objects), ϵ ≈ 0.16 in experiment 4 (10 recurring objects, 40 one-time objects), and ϵ ≈ 0.15 in experiment 5 (16 recurring objects, 64 one-time objects). Presumably, learning rates decrease as limited memory capacity is spread 'more thinly' over a larger number of objects.

At first glance, a second set of parameter values (ϵ ≈ 0.5 and *γ *≈ 0.25) accounts comparably well (and sometimes even better) for the experimental results (Figures 3df). However, a closer look reveals that this 'second' fit results from an intrinsic symmetry of the model: the overall learning rate is proportional to the product of ϵ and *γ *and thus may be matched equally well by (ϵ, *γ*) ≈ (0.25, 0.5) and by (ϵ, *γ*) ≈ (0.5, 0.25). In addition, low values of *γ *erode the recognition probability and thus provide an indirect way of adjusting the degree of context dependence. If one introduces a further parameter to modify the relative weights of current and previous objects, comparably good fits are obtained with high values of *γ *(not shown).

Finally, the results of experiment 3 are consistent with a learning rate of ϵ ≈ 0.14 and a wide range of recognition probabilities *γ*, with the best fit obtained for *γ *≈ 0.75. The comparatively low value of ϵ reflects the memory load, which was highest in this experiment (16 recurring and 112 one-time objects).

## Discussion

We have compounded the learning of multiple visual-motor associations in various sequential orders. In every trial, the rewarded response was fully predicted by a visible visual object. Additionally, however, the rewarded response was predicted to varying degrees by the visual objects of previous trials. Five experiments showed consistently that learning is accelerated when objects of previous trials provide a predictive temporal context.

In the first experiment, the trial sequence separated object-response-pairs with and without temporal context into distinct blocks, so that the difference was evident to observers. Reaction times were significantly shorter for objects with temporal context than for objects without temporal context, indicating that observers might have applied differential cognitive strategies. In the second experiment (and all others), trials with and without temporal context were intermixed, so that the difference remained concealed from observers. Reaction time patterns showed no evidence that observers allocated attentional/memory resources differentially to trials with and without temporal context. The third experiment raised task difficulty by doubling the number of visual objects (from 8 to 16), but confirmed the basic result: object-response-pairs with temporal context are learned faster than pairs without such context. In the fourth experiment, a partially predictive (20.3%) temporal context failed to accelerate associative learning. In the fifth and last experiment, the objects in successive trials formed ordered pairs, some predictive and others not. Only predictive pairings accelerated learning.

A number of previous studies have manipulated temporal context that *(i) *was irrelevant to the overt behavioral task and *(ii) *remained concealed from the observer. Typically, temporal context is altered by repeating a given set of trials in either fixed or random order.

In serial reaction time tasks [47], human observers respond as rapidly as possible to the locations of successive visual targets. After training, reaction times are faster when the target locations follow a repeating rather than a random pattern, which is taken as evidence of 'sequence learning' [31-33,48]. Importantly, observers do not have to be aware of the repeating sequence in order to benefit from it [49]. In serial button press tasks [36], non-human primates are presented with pairs of visual targets and learn to press two corresponding buttons in a particular order. Both within and between daily sessions, learning is facilitated when target pairs follow each other in a repeating rather than reversed or random order [50,51]. However, the animals do not seem to acquire choice responses for individual target pairs but rather motor sequences for 'hyper-sets' of several successive pairs [37,50].

In visual search tasks, human observers locate a single target (which is identified by certain distinguishing characteristics) among multiple distractors. Search performance benefits from the 'spatial context' that is provided by recurring distractor configurations [34]. Interestingly, observers are unaware of the repeating configuration and contextual learning depends on an intact hippocampus [35,52,53]. Similar benefits accrue from the 'temporal context' created when a fixed sequence of target locations is used in successive trials [54,55]. This temporal effect is also implicit and appears to be mediated by visual selective attention, in that observers learn to shift attention to the next target location predicted by contextual information. Finally, when different visual threshold discriminations (*e.g*., contrast, motion-direction) are compounded, visual learning accelerates significantly if different displays appear in a fixed (rather than random) temporal sequence [56]. It has been proposed that predictive temporal context may facilitate the activation of an appropriate visual template for each trial [57].

The present study differed from previous investigations in a number of ways. Firstly, it forced observers to become familiar with a number of initially unfamiliar fractal patterns. This emphasis on visual recognition was modeled on paradigms developed for behaving non-human primates [15,39,40].

Secondly, we ensured that observers associated individual fractal patterns with particular responses and foiled alternative strategies such as acquiring motor sequences that span several successive trials. We achieved this by keeping consistent sequences short (two trials in most experiments) and by intermixing trials with different temporal contexts. This sets our situation apart from serial reaction time [47] or serial button press tasks [36].

Thirdly, observers were able to attend fully to the sole visual object presented on each trial. This stands in contradistinction to visual search paradigms, where training improves performance mainly through the anticipatory guidance of visual selective attention [54,57,58].

Our behavioral results are quantitatively consistent with a model of reinforcement learning [44]. In this model, response choice is probabilistic but follows reward expectations, which are being accumulated in the form of 'action values'. The reinforcement rule increments (decrements) these 'action values' when a chosen response receives more (less) reward than expected. The key feature is that response choice is influenced by multiple 'action values', some attaching to the object of the current trial and others attaching to objects of preceding trials. Their effect is cumulative in the sense that the more 'action values' favor a particular response, the more likely this response is chosen. Accordingly, when successive objects appear in a consistent order, more than one 'action value' will favor the correct response, which will therefore be chosen more frequently.

The model accounts qualitatively and quantitatively for our behavioral observations, provided suitable values are chosen for learning rate ϵ and recognition parameter *γ*. The value of ϵ decreases as the number of fractal objects increases. The value is smaller than unity, which implies that observers concurrently acquire only a subset of stimulus-response pairings. Overall, the values of ϵ are consistent with the possibility that two to three pairings are being formed concurrently (*i.e*., at the ideal learner rate), while the remaining pairings are being ignored. The value of *γ *also decreases with the number of fractal objects, consistent with growing uncertainty about object identity.

In the present series of experiments, the task set remained stable in the sense that the same stimulus-pairings were rewarded throughout each trial sequence. However, stable tasks sets pose only a weak test of the model and its underlying assumptions. Far stronger tests can be devised with experimental designs that vary the task sets (*e.g*., task reversal). To illustrate this point, we outline a hypothetical experiment with variable task set:

Consider trials *i - *2, *i - *1 and *i *with stimuli *S*_*i*-2_, *S*_*i*-1_, *S*_*i *_and trial *i *with response *R*_*i*_. While the overt task is to acquire the pairing *S*_*i *_→ *R*_*i*_, the model additionally reinforces the pairings *S*_*i*-2 _→ *R*_*i *_and *S*_*i*-1 _→ *R*_*i*_. How will the model perform when either stimulus *S*_*i *_is replaced by  or response *R*_*i *_replaced by ? In the former case, two out of three pairings remain valid (*S*_*i*-2 _→ *R*_*i *_and *S*_*i*-1 _→ *R*_*i*_), so that predicted performance remains above chance. In the latter case, however, all pairings become invalid and predicted performance falls below chance. Accordingly, this hypothetical experiment would test the model's key assumption, namely, the reinforcement of pairings between past stimuli and present response (*S*_*i*-2 _→ *R*_*i *_and *S*_*i*-1 _→ *R*_*i*_).

Attractor network models of associative learning [25,26] are typically tested with electrophysiological recordings from behaving non-human primates [59-63]. However, behavioral observations from human observers can also furnish useful evidence, at least with respect to the more qualitative predictions of these theories. For example, behavioral experiments with sequences of self-similar images suggest that initially distinct classes of objects in associative memory become merged when exemplars of the two classes are repeatedly presented in the same temporal order [29,30]. This confirms the qualitative prediction that events occurring consistently in the same temporal order are eventually subsumed under one and the same event class in associative memory [27,28,64-66].

We have presented behavioral evidence that is consistent with another qualitative prediction of attractor network models, namely, the persistent representation of past events ('delay activity'). Patterning our behavioral situation on established paradigms of conditional associative learning, we have demonstrated that the presence of consistent temporal context significantly improves choice performance. This finding implies that not just the representation of a current event, but also the representations of past events, are reinforced during conditional associative learning.

## Conclusions

We believe that we have developed a promising novel approach for studying temporal context effects with human observers. Building on our current findings, we plan to characterize this persistent representation of past events more comprehensively in future experiments.

## Methods

A total of 38 female observers (mean age: 22.5; range: 20 - 32) were recruited from the university campus. All observers reported normal or corrected-to-normal visual acuity and were naive about the purpose of the experiment. Observers completed an informed-consent form approved by the ethics committee of the university.

### Apparatus and Stimuli

Highly distinguishable fractal objects with characteristic shapes and colors [39] were generated in Matlab using Psychophysics Toolbox (Brainard, 1997; Pelli, 1997) with an Apple computer (Dual 2 GHn PowerPC G5; 3.5 GB SDRAM, OS × 10.4). Stimuli were displayed on a grey background of an 22 inch Iiyama color monitor with a resolution of 1900 × 1200 pixels and a frame rate of 100 Hn. The display subtended 53° at the viewing distance of 50 cm. Fractal objects were presented foveally (diameter 4°) and four response options (grey disks of diameter 4°) appeared at 4° of eccentricity above, below, to the left and to the right.

### Task

Observers were instructed to learn to respond 'correctly' to each fractal object. It was explained that, for each fractal object, one of the four possible responses was 'correct', while the other three responses were 'incorrect'. Observers were told that they had to become familiar with and learn to recognize each fractal object and that they had to learn the 'correct' response of each object by trial and error. They were further told that there was no pattern or system that would enable them to predict which response a particular fractal object required. No mention of or reference to the sequence of trials and fractal objects was made.

### Procedure

Each trial comprised three phases (Figure 1a): 500 ms foveal presentation of a fractal object and four response options; 500 - 2000 ms response interval (terminated by the pressing of either ↑, →, ↓, or ← on the keyboard); 500 ms reinforcement (the chosen response option turned green if correct and red if incorrect). Blocks of 56 to 336 trials ('sequences') were performed without interruption. Each sequence used a new set of fractal objects, which had never been seen by the observer.

All sequences contained 'recurring objects', each of which appeared a certain number of times (6 to 14 times) during the sequence. At least 2 trials intervened between successive recurrences of the same object. Observers typically learned the correct motor response of recurring objects (although usually the sequence was terminated before performance reached 100% correct). With sufficiently long sequences observers do reach ceiling performance.

In experiments 2 to 5, sequences also contained 'one-time objects', which appeared only once per sequence. Obviously, observers could not hope to learn the 'correct' response for such objects. However, the results suggest that observers did not distinguish between recurring and one-time objects and expended comparable effort on both types of objects.

### Temporal context

We manipulated the sequence of objects to create a more or less predictive 'temporal context'. The current object completely determined the correct response (1 of 4 possible responses), corresponding to 2 bits of information. It is convenient to express the information provided by objects of previous trials about the correct response in the current trial as a percentage of 2 bits.

For example, the sequences in experiment 1 were either maximally deterministic or maximally variable. In the deterministic sequence, each object from an earlier trial was just as informative about the correct response in the current trial as the current object (100% information). In the variable sequence, objects from earlier trials carried no information about the correct response in current trials (0% information). The informativeness of the temporal contexts used in different experiments is summarized in Table 1. In experiments 2 to 5, different temporal contexts were intermixed in the same sequence: some objects were consistently embedded in a highly informative context (and other objects in a highly uninformative context). The types of temporal contexts used can be conveniently classified into types A to F.

#### Type A

objects were preceded by a one-time object and followed by one particular other recurring object (probability 100%). The temporal context provided by the preceding object was 0% in experiments 2, 3, and 5.

#### Type B

objects were preceded by one particular other recurring object (probability 100%) and followed by a one-time object. The temporal context provided by the previous object was 100% informative (experiments 2, 3, and 5).

#### Type C

objects were preceded (followed) by one-time objects (probability 50%) and by each of several other recurring objects (cumulative probability 50%). On average, the previous object was 2.8%, 0.5%, and 1.5% as informative as the current object (experiments 2, 3, and 4).

#### Type D

objects were preceded (followed) by one-time objects (probability 50%) and by one particular other recurring object (probability 50%). On average, the previous object was 20.3% as informative as the current object (experiment 4).

#### Type E

objects were preceded by a one-time object and followed by each of four other recurring objects (probability 25%). The previous object was 0% informative.

#### Type F

objects were preceded by each of four other recurring objects (probability 25%) and followed a one-time object. On average, the previous object was 0% as informative.

### Sequences

#### Experiment 1

Eight fractal objects appeared seven times each, in either a deterministic or a variable sequence. Both types of sequence were 56 trials long. In deterministic sequences, each object was preceded (followed) seven times (100% probability) by one particular of the other seven objects. In random sequences, each object was preceded (followed) once (14% probability) by each of the seven other objects. Target objects recur every 8 to 16 trials.

#### Experiment 2

Four recurring objects were used to form two consistent pairs (1 *- *2 and 3 *- *4), each of which appeared six times in the sequence. The 'predecessor' objects (1 and 3) were termed type A and the 'successor' objects (2 and 4) type B. Four additional recurring objects were used to form twelve random pairs (5 *- *6, 5 *- *7, ..., 8 *- *6, 8 *- *7), each appearing once per sequence (type C). Random pairs and consistent pairs were alternated and separated by 24 one-time objects to form sequences of 72 trials.

#### Experiment 3

Eight recurring objects were used to form four consistent pairs (1 *- *2, 3 *- *4, 5 *- *6, and 7 *- *8), each of which appeared fourteen times in the sequence. The 'predecessor' objects (odd numbers) were termed type A and the 'successor' objects (even numbers) type B. Eight additional recurring objects were used to form 56 random pairs (9 *- *10, 9 *- *11, ..., 16 *- *14, 16 *- *15), each appearing once per sequence. Random pairs and consistent pairs were alternated and separated by 112 one-time objects to form sequences of 336 trials.

#### Experiment 4

Five recurring objects were used to form five consistent pairs (1 *- *2, 2 *- *3, 3 *- *4, 4 *- *5, and 5 *- *1), each of which appeared eight times in the sequence. In contrast to earlier experiments, each object occurred in both the 'predecessor' and the 'successor' position. To mark this difference, we termed these objects type D objects. Five further recurring objects were used to form twenty random pairs (6 *- *7, 7 *- *8, ..., 8 *- *10, 9 *- *10), each of which appeared twice per sequence. As before, these objects were termed type C objects.

Random pairs and consistent pairs were alternated and separated by 40 one-time objects to form sequences of 120 trials.

#### Experiment 5

Eight recurring objects were used to form four consistent pairs (1 *- *2, 3 *- *4, 5 *- *6, and 7 *- *8), each of which appeared eight times in the sequence (type A and B). Eight further recurring objects were used to form sixteen semi-consistent pairs (9 *- *13, ..., 12 *- *13, 9 *- *14, ..., 12 *- *16), each of which appeared twice in the sequence. The 'predecessor' objects were termed type E (9, 10, 11, 12) and the 'successor' objects were termed type F (13, 14, 15, 16). Consistent and semi-consistent pairs were alternated and separated by 64 one-time objects to form sequences of 192 trials.

### Mutual information

We quantified the informativeness of temporal contexts in terms of mutual information. Assuming that responses are selected randomly (as is necessarily the case for unfamiliar objects), we computed the Shannon entropy *H *of the joint distribution of reward and motor response, conditional on the previous object(10)

where *p*(*r*_*t*_, *m*_*t*_*|s*_*t*-1_) is the joint probability of a reinforcement *r*_*t *_∈ {0, 1} and a motor response *m*_*t *_∈ {1, 2, 3, 4}, given that a particular object *s*_*t*-1 _occurred at the preceding trial *t - *1.

When temporal context is uninformative, a previous object does not restrict the set of possible next objects. In this case, the reward probabilities associated with the four responses are (1/4, 1/4, 1/4, 1/4). The full probability matrix for the joint occurrence of a particular response and a particular motor response is then

corresponding to an entropy of *H*_max _= 2.8113 *bit*. When temporal context is fully informative, the presence of a previous object completely determines the next object. In this case, the reward probabilities change to (1, 0, 0, 0) and the full probability matrix becomes

with an entropy of *H*_min _= 2 *bit*. The mutual information between the current object and the rewarded response is the difference between these values, or 0.8113 *bit*.

More generally, the informativeness of a previous object (trial *t - *1) about response-reward realization in the current trial was computed according to(11)

where the *H*_max _= 2.8113 *bit *and *H*_min _= 2 *bit*.

In the deterministic sequence of experiment 1, the previous object changes reward probabilities to (1, 0, 0, 0) (*H *= 2 *bit*), whereas, in the variable sequence, the previous object changes reward probabilities to (2/7, 2/7, 2/7, 1/7) (*H *= 2.7953 *bit*). Accordingly, in deterministic and variable sequences the previous object provides, respectively, 100% and 2.0% of the information that is provided by the current object. Conditioning on the preceding object alters the reward probabilities for type A and type B objects to (1/4, 1/4, 1/4, 1/4) and (1, 0, 0, 0), (entropy *H *= 2.8113 and *H *= 2 *bit*) respectively. Accordingly, the temporal context of type A and type B objects is 0% and 100%, respectively, as informative as the objects themselves. Conditioning on the predecessors of type C objects alters the average reward probabilities to (7/24, 7/24, 7/24, 3/8) in experiment 2 (*H *= 2.789 *bit*), to (15/56, 15/56, 15/56, 11/56) in experiment 3 (*H *= 2.8075 *bit*), and to (9/32, 9/32, 9/32, 5/32) in experiment 4 (*H *= 2.7992 *bit*), resulting in 2.8%, 0.5%, and 1.5% informativeness. Conditioning on the predecessors of type D objects in experiment 4 alters the average reward probability to (5/8, 1/8, 1/8, 1/8) with an entropy of *H *= 2.6463 *bit*. Thus, the predecessors are 20.3% as informative as the objects themselves. The predecessors of type E and type F objects in experiment 5 leave reward probabilities unchanged and thus are 0% informative.

## Authors' contributions

OHH designed and conducted all experiments, performed the analyses, worked out the reinforcement model, and wrote with JB the manuscript. JB conceived of the study and contributed to the reinforcement model. AW contributed to modeling issues. All authors read and approved the final version of the manuscript.

## Appendix: drift covariance matrix

In order to update the drift covariance matrix *P *^(*t*) ^we used the same equation as the one given in [46]:

where *I *is the identity matrix and **x **is the augmented stimulus vector. Once initialized (*P*^(0) ^= *I*), the drift covariance matrix *P*^(*t*) ^is computed recursively and an iteration takes place as follows:

1. 

2. 

3. 

4. 

5. 

The superscript *T *in *A*^*T *^indicates the transpose of *A *and (·)^-1 ^denotes the inverse matrix, which is the reciprocal in case of numbers.
